# Cytomegalovirus Retinitis in an ALL Child during Maintenance Therapy Treated Successfully with Intravenous Ganciclovir

**DOI:** 10.1155/2014/294238

**Published:** 2014-08-03

**Authors:** Hande Celiker, Ayse Karaaslan, Eda Kepenekli Kadayifci, Serkan Atici, Ahmet Soysal, Haluk Kazokoglu, Ahmet Koc

**Affiliations:** ^1^Department of Ophthalmology, Marmara University School of Medicine, Istanbul, Turkey; ^2^Department of Pediatrics, Marmara University School of Medicine, Istanbul, Turkey

## Abstract

*Purpose.* In here we described cytomegalovirus retinitis (CMVR) in 12-year-old male patient with acute lymphoblastic leukemia (ALL) who was on maintenance phase therapy. *Methods.* He was referred to our clinic for seeing of spots with the right eye for 3 days. At presentation, his best corrected visual acuity was 20/20 in the right eye and 20/20 in the left eye. Slit-lamp biomicroscopic examination of the anterior chamber of the left eye was within normal limits, whereas we observed 3+ anterior chamber cellular reaction in the right eye. On retinal examination, we found active retinitis lesions (cream-colored lesions associated with hemorrhages) and perivascular cuffing in the retinal periphery in the right eye. Left eye was normal. *Results.* On the basis of clinical picture, we made the diagnosis of CMVR in the right eye. Vitreous aspiration was performed and 23096 copies/mL of CMV DNA was detected by polymerase chain reaction (PCR) technique. The patient was successfully treated with intravenous ganciclovir for two weeks and discharged with oral valganciclovir prophylaxis. *Conclusion.* CMVR should be in mind in children with ALL on maintenance phase therapy even in those without hematopoietic stem cell transplantation. These patients can be treated successfully by intravenous ganciclovir alone.

## 1. Introduction

Cytomegalovirus (CMV) generally causes an asymptomatic or minimally symptomatic illness in immunocompetent patients; however, it is an important cause of morbidity and mortality in immunocompromised individuals [1, 2]. Depression of cell-mediated immunity due to immunodeficiency syndromes or secondary to immunosuppressive medications predisposes to symptomatic CMV infections such as retinitis, encephalitis, hepatitis, pneumonitis, and myocarditis [3]. In pediatric age group, acute lymphoblastic leukemia (ALL) is the most common hematologic malignancy and second most common malignancy overall in children [4]. Among the immunosuppressed states, cytomegalovirus retinitis (CMVR) was reported frequently in children with acquired immunodeficiency syndrome (AIDS) but rarely reported in other immunosuppressive conditions [5]. CMVR is more rarely in patients with ALL who did not receive hematopoietic stem cell transplantations (HSCT) [6]. However, CMVR is also quite rare in patients undergoing maintenance phase therapy (MPT) of ALL [7]. Herein, we report a case of bilateral CMVR in an ALL child in MPT who was not treated with autologous or allogenic HSCT before.

## 2. Case Report

**Figure 1:**
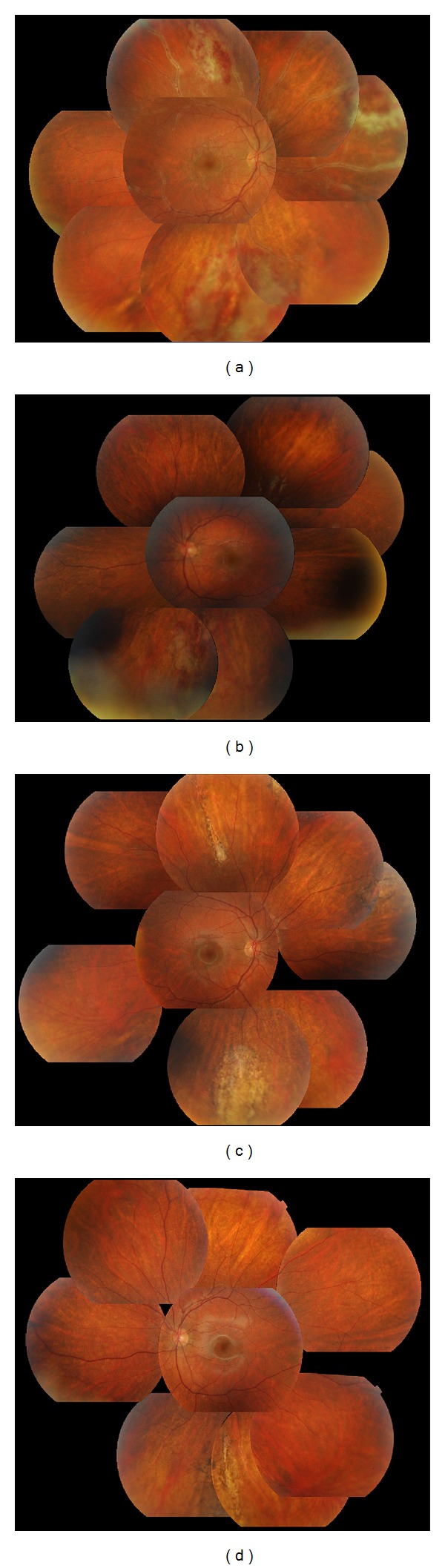
Color photographs of the right and left fundi. (a) Right eye fundus photograph at presentation showing active cytomegalovirus retinitis lesions (b). Left eye fundus photograph at the third day of treatment; the same retinal findings were also seen in the left eye but in a more limited pattern (c). And (d) two months after treatment, right and left fundus photographs show total resolution of active lesions, with the formation of chorioretinal scars, remission of retinitis.

A 12-year-old male was referred to our clinic for blurred vision in the right eye (RE) for 3 days. Systemically, he was suffering from ALL diagnosed >2 y ago; he had responded well to the induction therapy and obtained complete remission with treatment according to ALL-BFM 2003 protocol in maintenance phase therapy consisting of oral methotrexate and mercaptopurine only. His systemic workup to rule out CMV involvement of the other organs was negative. At the hospitalization of CMVR, hematologic workup revealed a white blood cell count of 3200/*μ*L and neutrophil count of 1600/*μ*L. At that time, his best corrected visual acuity (BCVA) was 20/20 in the RE and 20/20 in the left eye (LE). Slit-lamp biomicroscopic examination of the anterior chamber of the LE was normal, whereas we observed 3+ anterior chamber cellular reaction in the RE. On retinal examination, we found active retinitis lesions (cream-colored lesions associated with hemorrhages) and perivascular cuffing in the retinal periphery in the RE (Figure 1(a)). LE retina was normal. On the basis of clinical evidence, we made the diagnosis of CMVR in the RE. CMV IgM was positive and CMV DNA was also positive under 80 copies/mL in the blood sample. Intravitreal fluid was taken for examination of CMV DNA and 23096 copies/mL of CMV DNA was detected by PCR technique. Treatment with intravenous ganciclovir (10 mg/kg/d) was started immediately. At the third day of treatment, the same retinal findings were also seen in the LE but in a more limited pattern (Figure 1(b)). He was discharged after two weeks with oral valganciclovir prophylaxis (1800 mg/d × 15 d, followed by 900 mg/d × 1 m). On retinal examination, resolution of the active retinal lesions could be observed during the treatment period and a progressive pigment deposition was found around the lesions and developed into chorioretinal scarring (Figures 1(c) and 1(d)). The patient was followed up for one year and no recurrence was detected.

## 3. Discussion

Human CMV, also known as human herpesvirus 5, is a member of the herpesvirus family. CMVR is a major sight-threatening condition in neonatal CMV infection and immunocompromised children. To our knowledge, CMVR in ALL was only reported in six papers during maintenance chemotherapy [6–11]. There was no involvement of the posterior pole in our case; therefore, visual acuity was not affected.

Retinal involvement in acute leukemia may be due to different causes like direct leukemic infiltrates, vitreous and retinal hemorrhage caused by anemia, thrombocytopenia, or hyperviscosity [12]. They should be considered in the differential diagnosis. The diagnosis of CMV end organ disease is most definitively made by the detection of CMV DNA or RNA in tissues by in situ hybridization. However, in cases of retinitis, retinal biopsy is not often recommended because of the high risk of retinal detachment. In our case, CMV retinitis was suggested by the typical ophthalmoscopic appearance of hemorrhagic retinitis along with the concomitant rising of plasma CMV IgM and the detected CMV DNA by PCR in intraocular fluid sampling.

Prompt treatment is probably the most important prognostic factor; however, the optimal therapy route and drug for CMVR in children has not yet been found. Oral valganciclovir, intravitreal ganciclovir, intravenous ganciclovir, intravenous foscarnet, or intravenous cidofovir are the therapy regimens consistent with recommendations from the Centers for Disease Control and Prevention, the National Institutes of Health and the HIV Medicine Association guidelines [13]. In 80 to 90 percent of patients with AIDS, CMVR is treated with intravenous initial therapy (also known as induction therapy), which typically lasts 14 to 21 days, followed by maintenance therapy [14]. We have treated our patient only with intravenous ganciclovir successfully. We did not observe systemic complications of the intravenous ganciclovir such as neutropenia, thrombocytopenia, anemia, phlebitis, or gastrointestinal disturbances. Disadvantages of intravenous ganciclovir include decreased bioavailability in ocular tissues and a significant relapse rate; however, our patient was followed up to one year and no recurrence was found. CMVR is usually unilateral, but the patient is left untreated systemically; it may progress into the contralateral eye and result in severe visual loss. We treated our patient with systemic ganciclovir so we believe that we limited the spread to the other eye because retinal lesions of LE are milder than the RE in the patient. We think that this prevention would not be possible with intravitreal ganciclovir treatment alone.

The present case suggests that pediatric patients with ALL in the maintenance phase may be immunosuppressed and that clinicians should be alert to CMVR in these patients. Furthermore, CMVR should be kept in mind in children with ALL even in those without HSCT. Physicians should be vigilant to these rare occurrences. Early diagnosis and prompt treatment are important to preserve vision and prevent future visual morbidity.
